# What Is the Trajectory of Recovery in the Early Postoperative Period after the Big 3 Shoulder Surgeries? Comparative Analysis Using 3 Previous Prospective Studies

**DOI:** 10.3390/diagnostics14141532

**Published:** 2024-07-16

**Authors:** Du-Han Kim, Soon Gu Kim, Chul-Hyun Cho

**Affiliations:** 1 Department of Orthopedic Surgery, Keimyung University Dongsan Hospital, Keimyung University School of Medicine, 1035, Dalgubeol-daero, Dalseo-gu, Daegu 42601, Republic of Korea; osmdkdh@gmail.com; 2 Education Support Center, Keimyung University School of Medicine, 1035, Dalgubeol-daero, Dalseo-gu, Daegu 42601, Republic of Korea; sansori1112@dsmc.or.kr

**Keywords:** shoulder, rotator cuff repair, total shoulder replacement, reverse total shoulder replacement, outcome, recovery rate

## Abstract

(1) Background: The aims of this study were to compare serial changes in outcome measures in the early postoperative period after rotator cuff repair (RCR), anatomical total shoulder replacement (ATSR), and reverse total shoulder replacement (RTSR). (2) Methods: In total, 143 patients who underwent RCR (*n* = 47), ATSR (*n* = 46), and RTSR (*n* = 50) were included. The visual analogue scale (VAS) for pain, the activity of daily living (ADL) score, and the American Shoulder and Elbow Surgeons (ASES) score were completed. (3) Results: At 3 months, the recovery rate for the VAS pain score was 43.7% in the RCR, 89.1% in the ATSR, and 78.4% in RTSR. The recovery rate for the ADL score was 36.3%, 69.5%, and 76.4%. The recovery rate for ASES score was 40.9%, 79.5%, and 77.4%. For all outcome measures, a lower recovery rate was observed in the RCR group than in the ATSR and RTSR groups. At 6 months after surgery, the recovery rate for the VAS pain score was 69.9%, 100%, and 90.3%. The recovery rate for the ADL score was 66.8%, 92.8%, and 91.5%. The recovery rate for the ASES score was 68.7%, 96.5%, and 90.9%. (4) Conclusion: Compared with ATSR and RTSR, a slower recovery rate was observed for RCR, measured to be approximately 40% at 3 months and 70% at 6 months after surgery. Rapid improvement in pain and shoulder function was achieved after ATSR and RTSR, with a recovery rate of over 70% at 3 months and over 90% at 6 months after surgery.

## 1. Introduction

Rotator cuff repair (RCR), anatomical total shoulder replacement (ATSR), and reverse total shoulder replacement (RTSR) are the most common shoulder procedures. They have increased dramatically since the 2000s with the aging population [1,2]. The goal of these procedures is to improve the patient’s quality of life by improving shoulder function with substantial pain relief [3,4,5,6].

Numerous studies have demonstrated that RCR, ATSR, and RTSR produce favorable postoperative clinical results from short- to long-term follow-up periods [3,4,5,6,7,8,9,10]. However, a substantial period of time may be required after these surgeries for restoration of pain and shoulder function. Shoulder surgeons may sometimes underestimate the recovery timeframe in the early postoperative period with regard to various shoulder surgeries. In addition, patients may undergo surgery without having attained a clear understanding of the recovery process after index surgery. Moreover, patients often compare their expectations with those of other people who underwent a different surgical procedure [11]. Although RCR, ATSR, and RTSR are entirely different procedures with their own indications, surgeons may encounter cases involving overlapping indications [12,13,14]. For example, there are situations in which a certain procedure is the best option for patients with a massive rotator cuff tear (RCR vs. RTSR) or primary glenohumeral osteoarthritis without severe rotator cuff insufficiency (ATSR vs. RTSR). 

Early postoperative results after the index procedure have become the primary measure of patient satisfaction with the surgery [3]; thus, attaining a better understanding of the trajectory of recovery after various shoulder procedures is important for both surgeons and patients [11]. However, to date, there has been little concern regarding the recovery process for commonly performed shoulder surgeries. There is a paucity of available comparative data that may be helpful to surgeons and patients in the effort to understand how much improvement can be expected at each time point during the recovery process [15]. Therefore, we compared (1) serial changes in outcome measures in the early postoperative period after RCR, ATSR, and RTSR and (2) the rate of recovery along with the degree of improvement at each time point after index surgery. We hypothesized that the recovery pattern for RCR is slower than those for ATSR and RTSR.

## 2. Materials and Methods

### 2.1. Study Participants

This study was conducted using data from 3 previously reported prospective cohort studies [4,5,6]. Approval from the institutional review board of the authors’ affiliated institution was obtained with informed consent from all participants. An analysis of 143 patients, including 47 patients who underwent RCR for rotator cuff tear, 46 patients who underwent ATSR for osteoarthritis, and 50 patients who underwent RTSR for rotator cuff insufficiency, was performed. In total, 47 RCRs for rotator cuff tear (7 partial tears, 7 small tears, 17 medium tears, 7 large tears, 9 massive tears) were performed using an arthroscopic technique (*n* = 37) or an arthroscopic-assisted mini-open technique (*n* = 10) [5]. All procedures for ATSRs and RTSRs were performed using the deltopectoral approach [4,6]. The surgical technique and postoperative rehabilitation for each procedure were described in 3 previous studies [4,5,6].

### 2.2. Outcome Measures

Outcome measures included the visual analogue scale (VAS) for pain, the activity of daily living (ADL) score, and the American Shoulder and Elbow Surgeons (ASES) score. Pain intensity was measured via the VAS pain score, where a score of 0 points represents no pain and a score of 10 points represents unbearable pain. The ADL score contained 10 functional items that are shoulder-specific. Each of the 10 activities was scored on a scale from 0 to 3, depending on the difficulty encountered in performing the ADL. The ASES score is composed of the VAS pain score (50%) and the ADL score (50%) [5]. With assistance from a research coordinator, the patients completed all outcome measures before surgery and at 3, 6, and 12 months after surgery.

To examine the degree of recovery after RCR, ATSR, and RTSR, a comparison of estimated values for each time point (3- and 6-month) with those achieved at 12 months after surgery was performed [16]. Total improvement for each outcome measure was defined as the difference in values between before surgery and 12 months after surgery. The trajectory of recovery was regarded as the percentage of the total improvement achieved at 3 and 6 months after surgery [15].

### 2.3. Statistical Analysis

The Student *t*-test, chi-square test, and one-way analysis of variance (ANOVA) were used to determine the significance of differences between the 3 groups. The Scheffe post hoc test was used for precise determination of statistical difference between groups. Repeated measures of ANOVA were used to determine the time–effect and group-by-time interactions for each procedure through 12 months after surgery. The one-way ANOVA test was used for comparison of the difference in clinical scores between the 3 groups at each time point. Multiple comparison analysis was performed using the Scheffe post hoc test when a statistically significant group-by-time interaction was determined by one-way ANOVA. A two-tailed *p*-value less than 0.05 was considered statistically significant. Statistical analyses were conducted using SPSS 27.0 software for Windows^®^ (SPSS Inc, Chicago, IL, USA).

## 3. Results

The mean age of the patients who underwent RCR, ATSR, and RTSR was 57.1 ± 7.5 years (range, 43–75 years), 65.5 ± 10.0 years (range, 40–89 years), and 73.7 ± 5.9 years (range, 60–88 years), respectively. Differences in age (*p* < 0.001), duration of symptoms (*p* < 0.001), and sex (*p* = 0.018) were observed between the 3 groups. In the post hoc test, patients in the RTSR group were older than those in the ATSR group, and patients in the ATSR group were older than those in the RCR group. The duration of symptoms was longer in the ATSR group than those in the RCR and RTSR groups (Table 1). 

In all groups, a decrease in the mean VAS pain score and an increase in the mean ADL and ASES scores were observed after index surgery (all *p* < 0.001). Regarding serial changes in outcome measures, all clinical scores showed significant improvement (all *p* < 0.001). In the post hoc test for the analysis of the group-by-time interaction, the VAS pain score tended to be lower in the ATSR and RTSR groups than in the RCR group. The ADL score tended to be higher in the RCR and ATSR groups than in the RTSR group. The ASES score tended to be higher in the ATSR group than in the RCR and RTSR groups (Table 2).

Total improvement of the VAS pain score from before surgery to 12 months after surgery was 5.4 in the RCR group, 5.0 in the ATSR group, and 5.1 in the RTSR group. No differences were observed between the RCR and ATSR groups, RCR and RTSR groups, and ATSR and RTSR groups (all *p* > 0.05). Total improvement in the ADL score from before surgery to 12 months after surgery was 9.9 in the RCR group, 14.3 in the ATSR group, and 16.2 in the RTSR group. No difference was observed between the ATSR and RTSR groups (*p* > 0.05); however, differences were observed between the RCR and ATSR groups (*p* = 0.007) and the RCR and RTSR groups (*p* < 0.001). Total improvement in the ASES score from before surgery to 12 months after surgery was 43.8 in the RCR group, 48.6 in the ATSR group, and 52.8 in the RTSR group. No differences were observed between the RCR and ATSR groups and the ATSR and RTSR groups (all *p* > 0.05); however, a difference was observed between the RCR and RTSR groups (*p* = 0.008) (Table 2).

**Figure 1:**
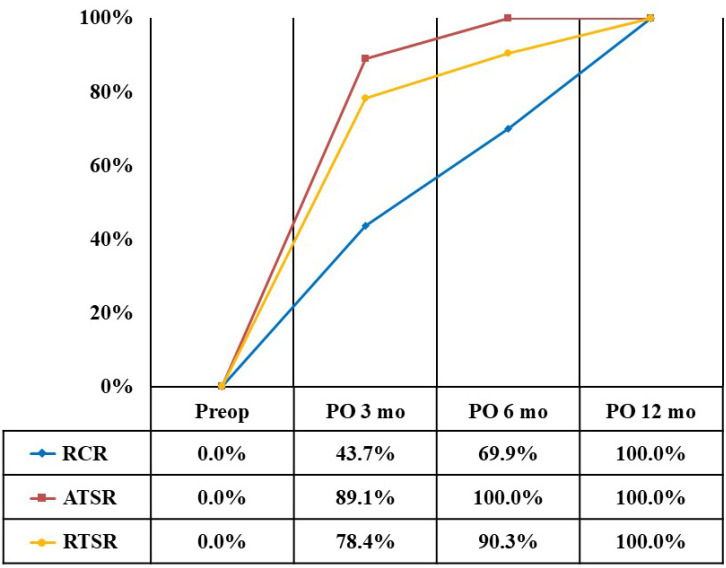
Recovery rate of VAS pain score.

**Figure 2:**
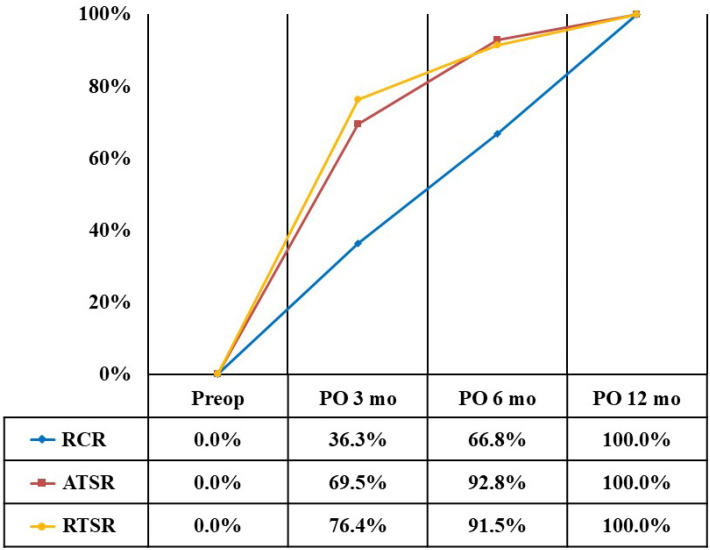
Recovery rate of ADL score.

**Figure 3:**
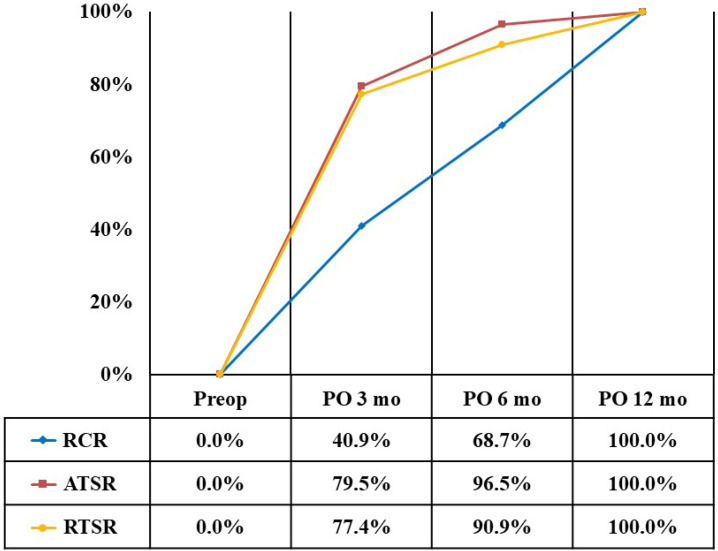
Recovery rate of ASES score.

At 3 months after surgery, the recovery rate for the VAS pain score was 43.7% in the RCR group, 89.1% in the ATSR group, and 78.4% in the RTSR group (Figure 1). The recovery rate for the ADL score was 36.3% in the RCR group, 69.5% in the ATSR group, and 76.4% in the RTSR group (Figure 2). The recovery rate for the ASES score was 40.9% in the RCR group, 79.5% in the ATSR group, and 77.4% in the RTSR group (Figure 3). In all outcome measures, lower recovery rates were observed in the RCR group than in the ATSR and RTSR groups (Table 3).

## 4. Discussion

This study was conducted to determine the rate of recovery along with the degree of improvement at each time point after commonly performed shoulder surgeries, which may be helpful to surgeons and patients in the effort to understand how much improvement can be expected at each time point in the recovery process.

According to the findings of this study, all clinical scores showed gradual improvement until 12 months after each procedure. In the post hoc analysis of group-by-time interaction, the VAS pain score tended to be higher in the RCR group than in the ATSR and RTSR groups. The ADL score tended to be higher in the RCR and ATSR groups than in the RTSR group. The ASES score tended to be higher in the ATSR group than in the RCR and RTSR groups. From before surgery to 12 months after surgery, total improvement in the VAS pain score was 5.4 for RCR, 5.0 for ATSR, and 5.1 for RTSR, with no difference between groups. Total improvement in the ADL score was 9.9 for RCR, 14.3 for ATSR, and 16.2 for RTSR, but was lower in the RCR group than in the ATSR and RTSR groups. Total improvement in the ASES score was 43.8 for RCR, 48.6 for ATSR, and 52.8 for RTSR but was lower in the RCR group than in the RTSR group. Considered together, these results indicate that RCR, ATSR, and RTSR can be regarded as effective procedures for eliminating pain and improving functional disability. In particular, ATSR and RTSR resulted in significant improvement in the ADL score as well as pain relief.

There is an interest in what the recovery process will be like and how much improvement can be expected at each time point in the recovery process after specific shoulder procedures. Patients undergoing RCR often inquire about how long it will take to recover from surgery in terms of both pain and shoulder function [15]. It is very important for the surgeon to counsel patients regarding the expected timeframe for postoperative recovery. Regardless of the repair technique, including arthroscopic, mini-open, or open procedures, RCR is known as a painful procedure with a long recovery period [3]. Kurowicki et al. [15] reported on the degree of recovery at each time point for pain and function for 627 patients who underwent arthroscopic RCR. They found that the plateau of maximum recovery occurred at 12 months after index surgery, with high satisfaction rates and approximately 75% of pain relief and 50% of functional recovery at 3 months. Cho et al. [3] reported that functional recovery based on clinical outcomes was approximately 60% of ultimate recovery at 3 months and approximately 75% of recovery at 6 months after RCR. Hughes et al. [17] reported that interval improvement in the constant score from before surgery to 3 months after arthroscopic RCR was just 3.2 points, with a decrease in muscle strength at 3 months postoperatively. However, interval improvement from 3 months to 6 months was 17.2 points, with significant difference. They emphasized that patients required assurance with regard to time and effort, focusing on physiotherapy until 6 months postoperatively. Levy et al. [16], who compared the speed of recovery following ATSR and RTSR, reported that rapid pain relief was achieved after both procedures. However, more consistent and effective recovery of pain, function, and shoulder rotation can be expected with use of ATSR [16]. Patients who underwent ATSR reached a consistent plateau for pain and function by 6 months and for shoulder elevation by 1 year. Patients who underwent ATSR had achieved 90% to 100% of functional improvement at 6 months postoperatively, whereas patients who underwent RTSR achieved 72% to 91% [16]. Grubhofer et al. [11] reported the speed of recovery after the most commonly performed shoulder surgeries, including ATSR, RTSR, arthroscopic RCR, and arthroscopic biceps tenodesis. They reported that the speed of recovery for all clinical scores was faster after ATSR at all measured time points and slowest after arthroscopic RCR and arthroscopic biceps tenodesis. At 6 months after surgery, the recovery rate for the ASES score was higher after ATSR and RTSR (96% and 85%, respectively) compared with arthroscopic RCR and arthroscopic biceps tenodesis (76% and 77%, respectively). However, due to the retrospective nature of the study and its high drop-out rate, they were not able to determine the recovery rate of clinical scores at 3 months after surgery. In the current study, a slower recovery rate was observed for RCR, with approximately 40% of total improvement at 3 months and 70% at 6 months after surgery. Rapid improvement in pain and shoulder function was achieved after ATSR and RTSR, with a recovery rate of over 70% at 3 months and over 90% at 6 months after surgery. At 3 months after surgery, lower recovery rates were observed for all outcome measures in the RCR group than in the ATSR and RTSR groups. At 6 months after surgery, the recovery rates for the ADL and ASES scores were lower in the RCR group than in the ATSR and RTSR groups. We believe that these findings provide useful information for surgeons and patients in the effort to understand the postoperative recovery process after shoulder surgeries.

This study has several limitations. First, the sample size was too small for an accurate comparison of 3 groups; however, data from our 3 previously reported prospective cohort studies, along with a power test, were used in this study. Second, the follow-up period (3, 6, and 12 months) was short. There was no follow-up evaluation at 2 years, which is usually recommended when conducting an outcome study. However, this study focused on serial changes in clinical scores in the early postoperative period. Several studies reported that maximal medical improvement was achieved at 12 months after RCR, ATSR, and RTSR [18,19,20]. Therefore, evaluation of sequential changes in this study might be sufficient. Third, preoperative demographic data, including age, sex, and duration of symptoms affecting postoperative clinical results, were heterogeneous among groups. However, a comparison of the postoperative clinical results after different shoulder surgeries was not the objective of this study. The rate of recovery with the degree of improvement were determined at each time point instead of direct comparisons of clinical scores after entirely different shoulder surgeries.

## 5. Conclusions

Compared with ATSR and RTSR, a slower recovery rate was observed for RCR, with approximately 40% at 3 months and 70% at 6 months after surgery. Rapid improvement of pain and shoulder function was achieved after ATSR and RTSR, with a recovery rate of over 70% at 3 months and over 90% at 6 months after surgery. These findings provide guidelines that the surgeons can use to inform patients about the expected postoperative recovery process during preoperative counseling for commonly performed shoulder surgeries.

## Figures and Tables

**Table 1:** Demographic data.

Variables	RCR ^a^ (*n* = 47)	ATSR ^b^ (*n* = 46)	RTSR ^c^ (*n* = 50)	Total (*n* = 143)	*p*-Value	Post Hoc Test
Age (year)	57.1 ± 7.5	65.5 ± 10.0	73.7 ± 5.9	65.6 ± 10.4	<0.001 *	a < b < c
Duration of symptoms	24.8 ± 35.7	91.4 ± 116.5	20.8 ± 21.0	44.8 ± 76.8	<0.001 *	a, c < b
Sex (male:female)	20:27	27:19	15:35	62:81	0.018 *	
Involved side (right:left)	32:15	32:14	30:20	94:49	0.564	

^a^ RCR, rotator cuff repair; ^b^ ATSR, anatomical total shoulder replacement; ^c^ RTSR, reverse total shoulder replacement. * Statistically significant, *p* < 0.05.

**Table 2:** Serial changes in clinical scores between groups.

	Group	Before Surgery		After Surgery							*p* Value	Post Hoc Test	η^2^
				At 3 Months		At 6 Months		At 12 Months					
		M ± SD	95% CI	M ± SD	95% CI	M ± SD	95% CI	M ± SD	95% CI				
VAS pain score	RCR ^a^	6.7 ± 1.6	6.3–7.4	4.3 ± 2.0	3.7–5.2	2.9 ± 2.1	2.1–3.8	1.3 ± 1.4	0.8–1.9	Group-by-time interaction	<0.001 *	a < b, c	0.063
	ATSR ^b^	6.2 ± 2.2	5.5–6.8	1.8 ± 2.5	1.0–2.5	1.2 ± 1.7	0.7–1.7	1.2 ± 2.1	0.6–1.8				
	RTSR ^c^	6.6 ± 2.1	6.0–7.2	2.6 ± 1.1	2.3–2.9	2.0 ± 1.3	1.6–2.3	1.5 ± 1.2	1.1–1.8				
ADL score	RCR ^a^	15.6 ± 7.3	12.7–18.0	19.2 ± 5.9	17.1–21.6	22.3 ± 4.9	20.1–23.9	25.5 ± 3.5	23.8–26.6	Group-by-time interaction	<0.001 *	c < a, b	0.108
	ATSR ^b^	11.0 ± 5.6	9.4–12.7	20.9 ± 6.9	18.9–23.0	24.2 ± 6.3	22.4–26.1	25.3 ± 5.3	23.7–26.8				
	RTSR ^c^	7.0 ± 5.1	5.6–8.4	19.3 ± 3.9	18.2–20.4	21.8 ± 4.2	20.6–23.0	23.2 ± 4.2	22.0–24.4				
ASES score	RCR ^a^	42.4 ± 16.5	34.9–48.0	60.2 ± 16.3	54.0–66.4	72.5 ± 13.5	66.4–77.2	86.2 ± 10.9	81.4–89.2	Group-by-time interaction	<0.001 *	a, c < b	0.089
	ATSR ^b^	37.4 ± 15.9	32.7–42.1	76.1 ± 20.0	70.1–82.0	84.3 ± 18.0	79.0–89.7	86.0 ± 18.1	80.6–91.4				
	RTSR ^c^	28.5 ± 15.6	24.0–32.9	69.3 ± 10.5	66.3–72.3	76.5 ± 12.6	72.9–80.1	81.3 ± 12.1	77.9–84.7				

VAS, visual analogue scale; ADL, activity of daily living; ASES, American Shoulder and Elbow Surgeons; ^a^ RCR, rotator cuff repair; ^b^ ATSR, anatomical total shoulder replacement; ^c^ RTSR, reverse total shoulder replacement. * Statistically significant, *p* < 0.05.

**Table 3:** Comparison of interval improvement for clinical scores between groups at each time point.

		VAS Pain Score			ADL Score			ASES Score		
		PO 3 mo	PO 6 mo	PO 12 mo	PO 3 mo	PO 6 mo	PO 12 mo	PO 3 mo	PO 6 mo	PO 12 mo
RCR vs. ATSR	M ± SD	2.4 vs. 4.4	3.8 vs. 5.0	5.4 vs. 5.0	3.6 vs. 9.9	6.7 vs. 13.2	9.9 vs. 14.3	17.9 vs. 38.7	30.1 vs. 46.9	43.8 vs. 48.6
	95% CI	1.8–3.4 vs. 3.4–4.7	2.9–4.6 vs. 4.0–5.3	4.8–6.1 vs. 4.5–5.8	0.5–7.3 vs. 10.7–14.0	3.9–10.0 vs. 13.1–16.5	7.9–11.9 vs. 14.6–17.7	11.1–27.8 vs. 36.1–45.6	23.3–37.7 vs. 43.2–52.8	38.9–48.6 vs. 48.2–57.5
	*p*-value	0.005 *	0.046 *	0.418	0.003 *	0.001 *	0.007 *	0.001 *	0.002 *	0.301
	*Cohen’s d*	−0.654	−0.450	−0.170	−0.676	−0.754	−0.572	−0.786	−0.715	−0.216
RCR vs. RTSR	M ± SD	2.4 vs. 4.0	3.8 vs. 4.6	5.4 vs. 5.1	3.6 vs. 12.3	6.7 vs. 14.8	9.9 vs. 16.2	17.9 vs. 40.8	30.1 vs. 48.0	43.8 vs. 52.8
	95% CI	1.8–3.4 vs. 3.5–5.3	2.9–4.6 vs. 4.1–5.8	4.8–6.1 vs. 4.0–6.0	0.5–7.3 vs. 7.5–12.3	3.9–10.0 vs. 10.9–15.5	7.9–11.9 vs. 11.8–16.7	11.1–27.8 vs. 31.4–45.9	23.3–37.7 vs. 39.7–54.1	38.9–48.6 vs. 40.4–56.8
	*p*-value	0.005 *	0.086	0.550	<0.001 *	<0.001 *	<0.001 *	<0.001 *	<0.001 *	0.008 *
	*Cohen’s d*	−0.629	−0.379	−0.122	−1.091	−1.054	−1.011	−1.056	−0.928	−0.553
ATSR vs. RTSR	M ± SD	4.4 vs. 4.0	5.0 vs. 4.6	5.0 vs. 5.1	9.9 vs. 12.3	13.2 vs. 14.8	14.3 vs. 16.2	38.7 vs. 40.8	46.9 vs. 48.0	48.6 vs. 52.8
	95% CI	3.4–4.7 vs. 3.5–5.3	4.0–5.3 vs. 4.1–5.8	4.5–5.8 vs. 4.0–6.0	10.7–14.0 vs. 7.5–12.3	13.1–16.5 vs. 10.9–15.5	14.6–17.7 vs. 11.8–16.7	36.1–45.6 vs. 31.4–45.9	43.2–52.8 vs. 39.7–54.1	48.2–57.5 vs. 40.4–56.8
	*p*-value	0.493	0.572	0.726	0.095	0.271	0.179	0.614	0.799	0.361
	*Cohen’s d*	0.141	0.116	−0.072	−0.350	−0.226	−0.227	−0.105	−0.053	−0.187

VAS, visual analogue scale; ADL, activity of daily living; ASES, American Shoulder and Elbow Surgeons; RCR, rotator cuff repair; ATSR, anatomical total shoulder replacement; RTSR, reverse total shoulder replacement. * Statistically significant, *p* < 0.05. At 6 months after surgery, the recovery rate for the VAS pain score was 69.9% in the RCR group, 100% in the ATSR group, and 90.3% in the RTSR group. The recovery rate for the ADL score was 66.8% in the RCR group, 92.8% in the ATSR group, and 91.5% in the RTSR group. The recovery rate for the ASES score was 68.7% in the RCR group, 96.5% in the ATSR group, and 90.9% in the RTSR group. Regarding the VAS score, a lower recovery rate was observed in the RCR group than in the ATSR group. Regarding the ADL and ASES scores, lower recovery rates were observed in the RCR group than in the ATSR and RTSR groups.

## Data Availability

The original contributions presented in the study are included in the article, further inquiries can be directed to the corresponding author.
